# YESS: A feasibility study of a supported employment program for youths with mental health disorders

**DOI:** 10.3389/fpsyt.2022.856905

**Published:** 2022-09-23

**Authors:** Nikki Ow, Kirsten Marchand, Krista Glowacki, Diana Alqutub, Steve Mathias, Skye P. Barbic

**Affiliations:** ^1^Department of Occupational Science and Occupational Therapy, Faculty of Medicine, The University of British Columbia, Vancouver, BC, Canada; ^2^Centre for Health Evaluation and Outcome Sciences, Vancouver, BC, Canada; ^3^Foundry Central Office, Vancouver, BC, Canada; ^4^Department of Psychiatry, Faculty of Medicine, The University of British Columbia, Vancouver, BC, Canada

**Keywords:** supported employment program, individual placement and support, youth mental health, health services, psychiatric rehabilitation

## Abstract

**Objective:**

In Canada, employment/education support is rarely embedded as a component of mental health service delivery. This study describes a supported education/employment program (SEP) that integrates both clinical and community mental health services. The main objectives were to estimate the feasibility of a 5-week SEP among youths aged 17–24 with mental illness and to estimate the extent to which participation in this program improved employment and mental health outcomes.

**Methods:**

This was a single cohort study. Feasibility outcomes assessed were demand, acceptability, practicality, integration, adaptation, and effectiveness. These were assessed through recruitment and retention rates, recording patterns of missing data, and examining differences between completers and non-completers. Appropriateness of the outcome measures was assessed through the strength of the association between the outcome measures at baseline. Effectiveness of the program was assessed through employment and as measured using MyLifetracker (MLT), Satisfaction with Life Scale (SWLS), and the Canadian Personal Recovery Outcome Measure (CPROM).

**Results:**

A total of 110 youths with a mean age of 20.6 (SD: 2.2) were recruited. At 5 weeks, 82 (74.5%) of participants remained in the program. Of the people who completed the program, 56.1% were women, 76.6% were in stable housing and 64.1% had depression. Approximately 60% of non-completers used two or more services and were in at-risk housing. More than 25% of participants improved on the patient-reported outcomes. Scores on these measures were moderately to highly correlated with each other. Employment rates varied and corresponded to the waves of the COVID-19 pandemic in Canada.

**Conclusion:**

Results showed that this program was feasible and there was high demand for SEP during the COVID-19 pandemic but gaining employment remained difficult. Educational or employment outcomes, measured over a short period, may not be adequate. Instead, individualized and patient-reported outcome measures may be more appropriate for SEP programs.

## Introduction

Employment and income have been identified as important social determinants of health in Canada (1) and by the World Health Organization (2, 3). *Employment* shapes income, living conditions and access to resources, affects psychological functioning and identity, and influences health-related behaviors such as quality of diet, engagement in physical activity, tobacco use, and substance dependence (3, 4). In Canada, ~70–90% of people with serious mental illness are unemployed in Canada, and this trend begins early in life (5, 6). For youths aged 19–24 years, only 48% of those with mental illness report being employed or in school, compared to 87% without a mental illness (5, 7). In a country where mental illness affects ~1 in 4 youth aged 15–24, these are worrying trends (8). The additional burden of being unemployed can bring about further stress and anxiety. On the other hand, being employed can bring about recovery and health benefits among people with mental illness.

Most people with mental illness want to work but are often unable to find suitable and meaningful work (9). Challenges are even greater for those who have not completed their education and/or developed effective work habits and skills to cope with their illness (5, 10). Many barriers to gainful employment exist for youths with mental illnesses (11). This problem has been exacerbated by the COVID-19 pandemic. Since the start of the pandemic, Canada's unemployment rate among youths has declined, with <50% employed in 2020 (12). Hence, in parallel with high-quality mental health care, earlier intervention and more effective employment services are needed to enable youths with mental health conditions to successfully transition to early adulthood and employment or post-secondary education.

In Canada, many interventions to support youths with mental health conditions in obtaining employment are based on the Individual Placement and Support (IPS) model (13, 14). IPS is an evidence-based supported employment model for people with mental illness (15), designed to achieve employment in mainstream competitive jobs, either part-time or full-time (16). This approach is in contrast to traditional vocational approaches, which typically employ people in sheltered workshops or other non-competitive jobs, or provide extended periods of prevocational training (17, 18). Research has shown that IPS produces better competitive employment outcomes for people with mental illness compared to alternative vocational programs (19, 20). However, youth-specific mental health care and employment services in Canada are often provided by separate agencies, and employment/education support is rarely embedded as a component of clinical service. Hence, little is known about the effectiveness of integrating supported employment models into integrated health teams that serve youths with a range of mental health conditions. This study describes the feasibility of a novel intervention that builds on the potential benefits of a supported work/education model that is embedded within both clinical and community services.

This study had two objectives: (1) to estimate the feasibility of the Youth Employment Skills Strategy (YESS) program among youths aged 17–24 years with mental illness, and (2) to estimate the extent to which participation in the YESS program improved employment rate and mental health outcomes in youths after 5 and 16 weeks. Results of this study will help inform the planning and implementation of a larger scale supported employment program across an integrated youth service network in British Columbia (BC).

## Materials and methods

### Study design

This study was a 16-week single cohort study assessing the effectiveness of the YESS program. This paper presents results based on data collected at three timepoints: baseline, 5-, and 16- weeks.

### Funding

This study was funded by a Service Canada grant to increase employment in youths with MHSU disorders. The funding was specifically catered to youths aged 17–24 years old who were neither employed nor in educational or training programs and the main outcome for this program was employment as set by the funder.

### Settings and participants

This study was conducted in Vancouver, BC, Canada. The YESS program was conducted is an integrated youth service centre that provides psychiatric care, psychosocial rehabilitation and other services for youths aged 17–24 with mental health and substance use (MHSU) challenges in the Greater Vancouver Area. In this centre, all participants have access to mental health support services like counseling, substance use support, peer support, and psychiatric services.

#### Recruitment, screening and consent

Participants were recruited at the centre. Eligibility criteria included the following: interested in pursuing employment, not employed or in school for <15 h per week, aged 17–24 years, and diagnosed with a mental illness (defined as one or more of the following: major depressive disorder, bipolar disorder I, II, anxiety disorder, schizophrenia spectrum disorder, schizoaffective disorder, delusional disorder; and psychotic disorder; not otherwise specified) or substance use disorder. Eligible participants were contacted by center staff. All participants who expressed interest were recruited into the study since participation in the research study was a requirement for the YESS program. Recruitment occurred over a 13-month period from November 2019 to December 2020. During the COVID-19 pandemic, recruitment was done online through advertisements on Google and Instagram. Participants were screened for YESS program eligibility by staff at the center and a research coordinator. All participants provided written informed consent prior to any research procedures taking place. The study was approved by the University of British Columbia's Behavioral Research Ethics Board (H21-01510).

### Sample size

For this feasibility study, we set out to recruit 100 participants for the 16-week duration of the study due to staff capacity and logistical reasons. After factoring in a 10% drop-out rate, we aimed to recruit 110 participants.

### Description of the intervention

The TiDier guidelines were used to describe this intervention (21). The YESS program was an integrated supported employment and education intervention program was developed based on IPS principles. The YESS program offered health, social, and employment skills education in small groups, one to one consultation and specific job training courses to youths aged 17–24 with MHSU disorders. The focus of this supported employment program was on developing youths educational qualifications, work interests and work readiness and not on disability status, making it different from other supported employment programs. The team primarily comprised of two IPS specialists - an occupational therapist (OT) and a vocational rehabilitation counselor, a peer support worker, and a program coordinator. The program coordinator was mainly in charge of building relations with potential employers, promotion of the YESS program and carrying out the screening of potential participants. All participants had one-to-one sessions with either the OT or the vocational counselor to identify goals and develop an individual customized employment or education plan. Both the OT and vocational counselor carried out the various training courses or facilitated the workshops with the invited speakers. The OT carried out vocational assessments when needed while the vocational counselor provided vocational counseling to participants. The peer support worker provided support to participants when needed and assisted in their job search. Other professionals like social workers and psychiatrists were involved when needed. Other than the team members, all other health care professionals were employees of the integrated youth service center.

#### Processes involved

Upon recruitment into the program, the team first assessed the participant's employment history, strengths, interests, and skills. When these were identified, the team worked together with the participant to explore different career options and select the relevant training courses needed for gainful employment. For participants who did not have a specific job in mind, the team worked with them to help with the job search and job application.

Job matching to specific industries was facilitated by the IPS specialists. To enable a successful job matching, team members first did an environmental scan to identify and engage local businesses, educational and training programs in the Greater Vancouver Area. They also worked with the staff at the youth center to identify local businesses that had existing relationships with the centre. In addition, members of the team attended job fares, and employment programs networks to leverage resources. An updated list of job opportunities in the Greater Vancouver Area was sent to all participants on a bi-weekly basis.

#### Duration and description of program

The whole duration of the YESS program was 5 weeks, with follow up support up to 16 weeks. As youths were recruited throughout the year, the 5-week intervention was offered every 3 months. During the first 5 weeks, the primary intervention was one to one work readiness and job matching support provided by the IPS specialists. Youth could also participate in small group training workshops if they were interested. Examples of these small group workshops include: Understanding Emotions, Mindfulness, Coping Strategies, Communication Skills, Motivation, Resumes and Job Searching techniques, Interview Skills, Disclosure and Accommodations, and Preparing for Workplace. These workshops were held twice a week and each session was an hour long. The workshops were delivered in person in small groups, however this format changed to virtual workshops due to the COVID-19 restrictions in March 2020 (hosted virtually through Microsoft Teams). One to one support was also changed to virtual sessions during the pandemic. Some participants also obtained additional training (e.g., food safety, first aid, barista certification) as needed for their desired job. These courses ranged from a few days to more than a week. Lastly, participants could also do on job placements from 9 A.M. to 3 P.M. as part of the YESS intervention.

After 5 weeks of training courses and job placements, participants were encouraged to apply for jobs. During the 11-week follow-up period, team members continued to provide one to one support to all participants when needed, in various areas like job application or peer support.

### Data collection

Demographic data were collected at baseline. Data collection was done in-person before the pandemic *via* paper and pencil or using iPads provided at the centre. However, during the COVID-19 pandemic, due to lockdown restrictions, data was collected *via* an online survey. All data was entered into a secure database. Data collected were self-reported and included gender identity, age, ethnicity, sexual orientation, diagnosis, past educational attainment, housing status, current employment, and school attendance in the last 6 months.

### Measurement strategy

#### Feasibility outcomes

The aim of this feasibility study was to contribute evidence toward the implementation of a larger scale supported employment program in BC, we hoped to understand the factors that influenced fidelity and implementation of the YESS program (22). Therefore, our methods were to assess recruitment, retention and procedures of the program (22) and we focused on the following feasibility outcomes as outlined by Bowen et al. (23): Demand, acceptability, practicality, implementation, integration, and adaptation. Demand and acceptability of the program were assessed through recruitment, retention, and attrition rates, respectively. Specifically, the total number of participants who completed 5 weeks of the program, and the number of dropouts were recorded. Practicality of program was assessed by recording patterns on missing data on all study variables and implementation was assessed by examining the results of the appropriateness of the outcome measures. This metric was selected because the YESS program applied a routinely collected self-reported demographic questionnaire from the integrated youth service center as its main clinical data source. As this questionnaire comprehensively captures youths' self-reported demographic and health outcomes data, the pattern of missing data was inspected to determine this questionnaire's appropriateness and if the data collected was sufficient to analyze program outcomes. The appropriateness of the outcome measures was assessed through the strength of the association between the outcome measures at baseline. Missing data on outcome measures also informed the appropriateness of the outcome measure. These data informed the implementation and sustainability of the program. To assess the level of integration into the existing network of services, any changes made to the YESS program during this study period were also recorded. Recruitment through the COVID-19 pandemic restrictions was recorded as an assessment of adaptation. Adaptation was also assessed through examining the difference between participants who have completed the program and non-completers.

To estimate the extent to which participation in the YESS program improved employment rate and mental health outcomes, we looked at limited efficacy testing (22, 23). Limited-efficacy testing is the testing of the intervention in limited way (23) and was assessed by estimating the change in outcome measures at five and 16 weeks.

#### Outcome measures

The primary outcome measure was the proportion of youth employed (self-reported full-time or part-time employment or casual work) after 5 weeks of intervention and at 16 week follow up.

Secondary outcome measures included five patient reported outcome measures measured at 5 weeks and 16 weeks:

1. The Canadian Personal Recovery Outcome Measure (CPROM), a 30-item scale developed for Canadians with mental illness living in the community, total scores on the CPROM were calculated by dividing the sum of all scores by 4, with higher scores reflecting better recovery (24).
2. The MyLifeTracker (MLT), a 5-item mental health outcome measure designed for routine use. The five items cover five domains: general health, day to day activities, relationships with friends, relationship with family and coping. Total MLT scores were calculated by averaging across the five items, ranging from 0 to 100, with a higher score indicating a higher quality of life (25).
3. The Satisfaction with Life Scale (SWLS), a 5-item scale measuring life satisfaction. Responses are denoted on a 7-point Likert scale ranging from strongly agree to strongly disagree. Total scores are calculated by summing all scores on the five items, with higher scores reflecting higher satisfaction (26).
4. The General Anxiety Disorder (GAD-7), a 7-item screening tool for assessing generalized anxiety disorder. Each item is assigned a score of 0, 1, 2, and 3, corresponding to the response categories of “not at all,” “several days,” “more than half the days,” and “nearly every day,” respectively, total scores are obtained by adding together the scores for the seven questions (27).
5. The Physical Health Questionnaire (PHQ-9), a 9-item scale that measures depressive symptoms. Each of the nine items are scored from “0” (not at all) to “3” (nearly every day). Total scores are calculated by summing all scores on the nine items (28).

### Statistical analysis

Distributional parameters were used to summarize baseline demographic variables. To estimate the limited efficacy of the YESS program among youths with mental health conditions, mean scores of all outcome measures at baseline and at week 5 were presented. Proportions of people who improved on these outcomes were presented at 5 and 16 weeks. Improvement on all measures refers to any increase in scores greater than the minimally clinically important change (MCID) score. For the GAD-7 and the PHQ-9, an improvement of four and five points, respectively, is clinically significant (29, 30). For the other measures, the MCID scores were not available and thus, a change of half standard deviation or more was recorded. To assess if the outcome measures were appropriate, Pearson correlation was conducted between the baseline measures. Correlations between demographic variables and outcomes measures were also carried out to identify possible predictors. Pearson correlation was conducted for continuous variables (age and outcome variables), rank biserial correlation was used for ranked binary and continuous variables (highest education and outcome variables), biserial correlation was used for binary and continuous variables (at-risk/not at-risk housing and outcome variables) and polyserial correlation was used for discrete variables and continuous variables (gender and outcome variables).

To assess acceptability and demand of the program, the proportion of people who completed the program was computed. To assess adaptation, demographic characteristics of people who completed the program at week 5 were compared with non-completers. Adaptation of the program under COVID-19 restrictions was assessed by plotting the proportion of people employed over 1 year along with the timings of COVID-19 provincial-wide and nation-wide restrictions. Patterns of missing data in demographic and outcome variables were also identified.

## Results

**Figure 1:**
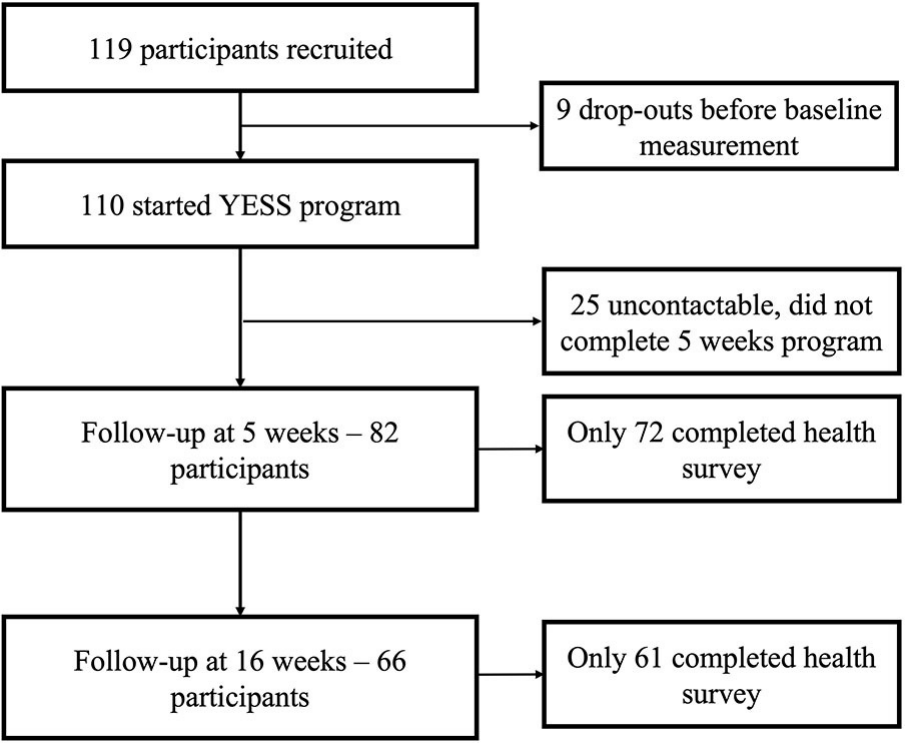
Study process.

A total of 119 participants were recruited in the study but nine participants dropped out before baseline measurement (see Figure 1). Participants' mean age was 20.6 (SD: 2.2) (Table 1). Close to half of participants were white (47.3%), and most identified as women (52.3%). More than 60% of the participants reported that they had depression, 68.2% had completed secondary education and 76.6% had stable housing.

**Table 1:** Baseline characteristics of participants.

	***n* = 110 (%)**
Mean age (SD)	20.6 (2.2)
**Gender**	
Woman	58 (52.7)
Man	38 (34.5)
Other	14 (12.7)
**Ethnicity/cultural background**	
Asian and Pacific Islander	19 (17.3)
African	<5
Caribbean	<5
Hispanic/Latino	<5
Indigenous	10 (9.0)
Middle Eastern	<5
White/European descent	52 (47.3)
Mixed ethnicity	17 (15.5)
Participants with depression	63 (57.3)
**Highest educational level**	
Secondary education incomplete	35 (31.8)
Secondary education completed	75 (68.2)
**Living situation** ^**$**^	
I am homeless/couch surfing	<5
I live with someone else (i.e., parents, friends)	52 (52.5)
I live on my own in my apartment	15 (15.2)
Supported housing/single room occupancy	31 (31.3)

$ missing data. At risk housing includes people who are homeless, couch surfing or living in supported housing or single room occupancy.

Table 2 shows the mean scores on all outcome measures at baseline, 5 weeks, and 16 weeks. At 5 weeks, 13 people were employed either part-time or full-time, and 22 people were employed at 16 weeks, an ~18% increase. Mean scores on all outcome measures improved across all timepoints. Table 3 shows the proportion of people who improved across all measures and all timepoints. At 5 weeks, 44.4% of people showed an improvement of more than half a standard deviation on the SWLS, followed by the MLT (38.9%). On the GAD-7, 33.3% of people improved by more than 4 points at 5 weeks. On the PHQ-9, 23.6% of people improved by 5 points or more. At 16 weeks, the proportion of people who improved were generally stable, with the biggest proportion of people who improved recorded on SWLS (37.7%), followed by 34.4% on the CPROM.

**Table 2:** Mean scores of outcomes measures at baseline, 5 and 16 weeks.

**Outcome**	**Baseline, mean**	**5 weeks, mean**	**16 weeks, mean**
**measures**	**(SD)**	**(SD)**	**(SD)**
	**(*n* = 110)**	**(*n* = 72)**	**(*n* = 61)**
Employed, n (%)^$^	0	13 (18.1)	22 (33.3)
MLT	54.2 (19.1)	61.7 (15.1)	57.4 (17.9)
GAD-7^%^	11.5 (5.7)	10.5 (4.8)	10.7 (4.9)
PHQ-9^%^	13.6 (6.7)	12.0 (5.2)	12.4 (5.5)
SWLS	16.9 (7.1)	19.1 (6.7)	20.6 (8.4)
CPROM	(5.6)	17.7 (4.6)	17.5 (5.2)

MLT, MyLifeTracker; GAD-7, The General Anxiety Disorder Scale; PHQ-9, The Physical Health Questionnaire; SWLS, The Satisfaction with Life Scale; CPROM, The Canadian Personal Recovery Outcome Measure.

$ Data available for 82 participants at 5 weeks and 66 participants at 16 weeks.

% For the GAD-7 and PHQ-9, lower scores indicate improvement, for all other measures, higher scores indicate better function.

**Table 3:** Proportion who improved from baseline at 5 weeks and 16 weeks.

**Outcome measures**	**5 weeks (*n* = 72)**	**16 weeks (*n* = 61)**
Employed (%)	13 (15.8)	22 (33.3)
MLT	28 (38.9)	20 (32.8)
GAD-7^$^	24 (33.3)	17 (27.9)
PHQ-9^$^	17 (23.6)	15 (24.6)
SWLS	32 (44.4)	23 (37.7)
CPROM	20 (27.7)	21 (34.4)

$ For the GAD-7 and PHQ-9, results represent the proportion of people whose scores changed by four and five points, respectively. For all other measures, change is marked by an improvement of $\frac{1}{2}$ SD or more.

The correlations between demographic variables and outcome measures are presented in Table 4. The strongest correlations were found between the GAD-7 and the PHQ-9 (0.78), the PHQ-9 and the CPROM (-0.71) and the CPROM and MLT (general wellbeing) (0.71). The strength of the correlations between employment and other variables ranged from 0 to 0.17. The strength of the correlations between demographic variables and outcome measures ranged from 0 to 0.31, with the strongest correlation found between gender and MLT (coping).

**Table 4:** Heatmap of correlations.

**Variables**	**Age**	**Gender**	**Type of housing**	**Highest education**	**No. of services**	**GAD7**	**PHQ9**	**SWL**	**CPROM**	**Employment**
Age	1									
Gender		1								
Type of housing			1							
Highest education	0.36		−0.10	1						
No. of services used	0.15		0.29	0.04	1					
GAD7	0.00	−0.10	−0.06	−0.04	−0.12	1				
PHQ	−0.01	−0.18	0.03	−0.08	−0.05	0.78	1			
SWL	−0.04	0.07	−0.01	−0.15	0.14	−0.45	−0.49	1		
CPROM	0.00	0.25	0.10	−0.11	0.13	−0.57	−0.71	0.67	1	
MLT	−0.009	0.24	0.04	−0.06	0.12	−0.60	−0.70	0.61	0.76	−0.06
Employment at 5 weeks	−0.17	−0.10	−0.10	0.04	−0.14	0.05	−0.01	0.00	0.05	1

The darker the color indicates the stronger the correlation.

**Figure 2:**
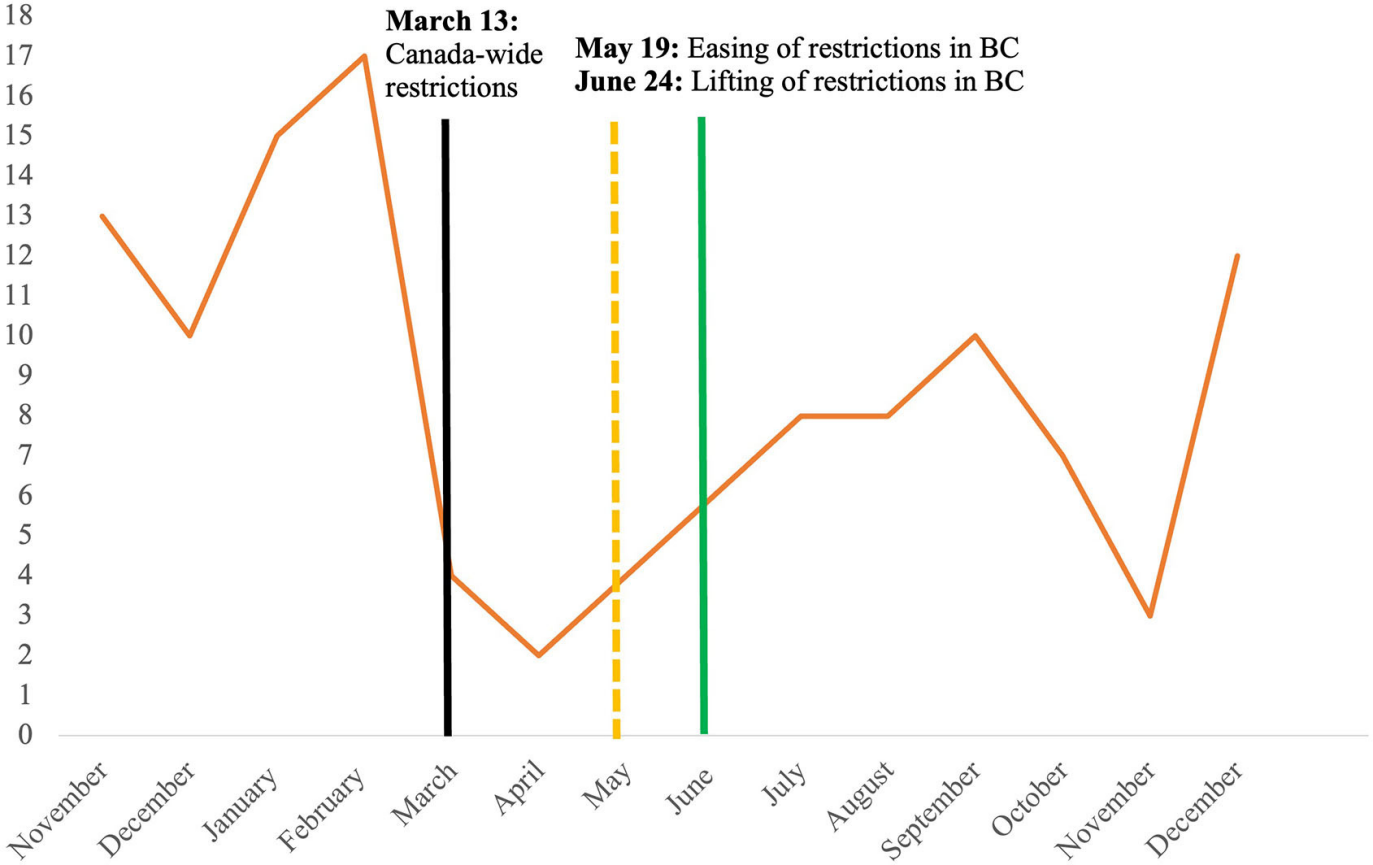
Recruitment rate from November 2019 to December 2020.

Figure 2 illustrates the number of participants recruited from November 2019 to January 2021. Over the study period, recruitment rates corresponded with the restrictions imposed by the federal and provincial government in Canada and British Columbia. From April 2020, the YESS program had to be adapted due to the restrictions. Recruitment was conducted virtually through advertisements with links to program website online, and the program pivoted to be fully online within 2 weeks. The average number of referrals from online recruitment was 83 per month from May to November 2020.

Altogether, 82 (74.5%) participants completed the whole 5-week program. Table 5 shows the demographic characteristics of completers and non-completers at 5 weeks. There were no differences between these two groups across all outcome measures at baseline. However, at 5 weeks, of those who completed the program, there were more people who identified as women (56.1%), and who reported living in stable housing (76.6%) and having depression (64.1%). More than 60% of non-completers used 2 or more services and were in at-risk housing.

**Table 5:** Comparison of baseline data of completers and non-completers at 5 weeks.

	**Completers**		**Non-completers**	
	**(with 5-week data)**		**(without 5-week data)**	
	***N***	**Mean (SD)**	***N***	**Mean (SD)**
Age	82	20.4 (2.3)	28	21.2 (1.8)
Female, n (%)	82	**46 (56.1)**	28	**12 (42.9)**
With depression, n (%)	78	50 (64.1)	23	13 (56.5)
**Number of services used**	77		21	
≤ 1		**43 (55.8)**		**8 (38.1)**
≥ 2		**34 (44.2)**		**13 (61.9)**
**Highest education level, n (%)**				
Completed secondary education	81	55 (67.9)	28	20 (71.4)
**Living situation, n (%)**				
At risk housing^$^	77	**18 (23.4)**	22	**15 (68.1)**
GAD7	81	12.5 (5.3)	28	13.8 (7.6)
PHQ9	81	13.2 (6.0)	27	10.4 (6.3)
SWLS	82	16.6 (6.7)	28	17.3 (8.0)
CPROM	80	17.6 (5.3)	28	16.5 (6.2)
**MLT**				
General wellbeing	81	50.7 (23.1)	28	50.0 (24.3)
Day to day	81	53.5 (22.3)	28	50.4 (25.6)
Relationships	81	62.9 (25.9)	28	63.2 (27.5)
Coping	81	51.3 (24.1)	28	53.9 (29.6)
Family relations	81	54.1 (29.9)	28	48.2 (29.8)
Total	73	54.3 (18.2)	28	53.1 (21.8)

$ At risk housing includes people who are homeless, couch surfing or living in supported housing or single room occupancy. The bold values indicate the difference greater than 10% between completers and non-completers.

**Figure 3:**
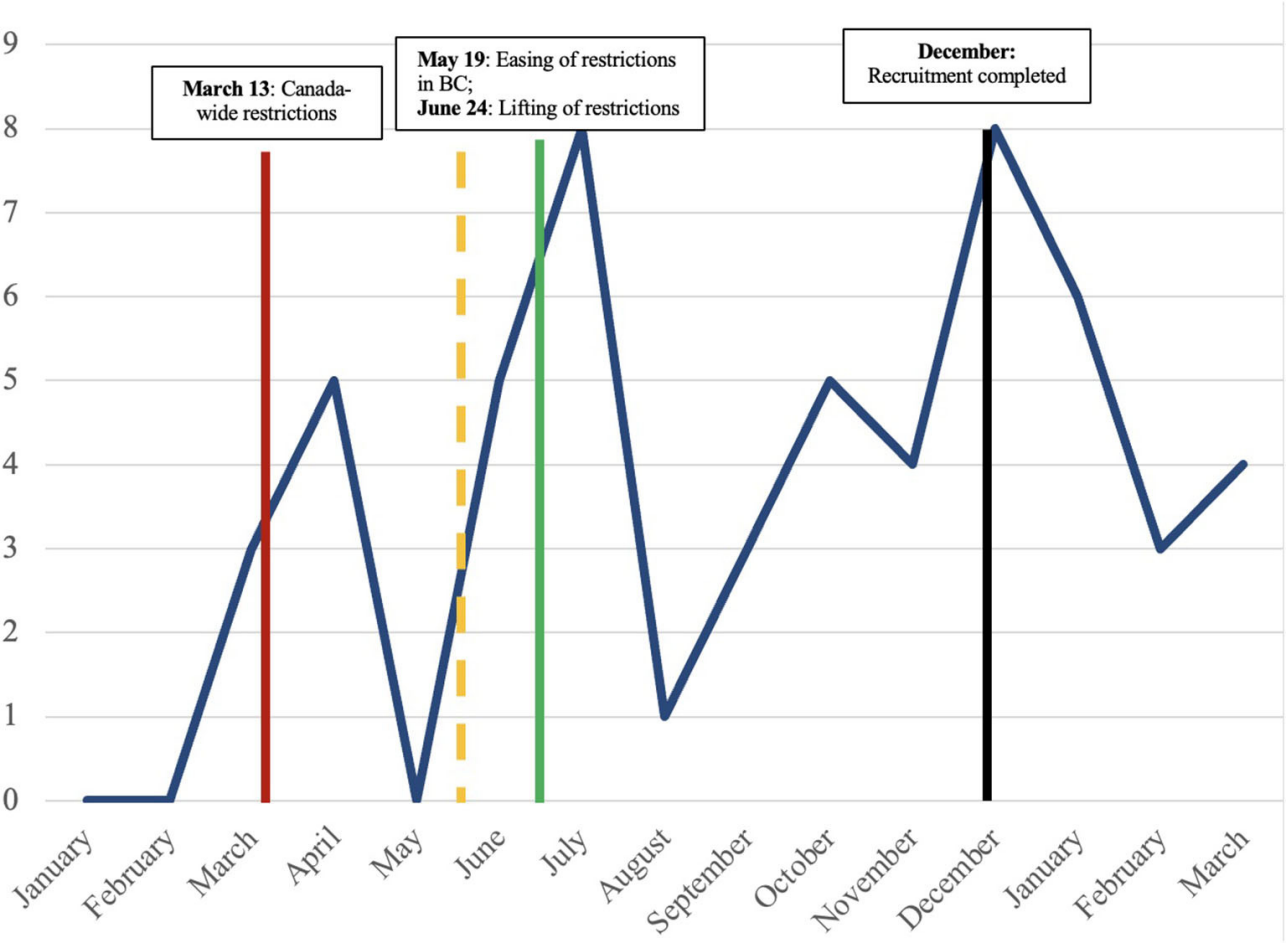
Number of participants employed from January 2020 to March 2021.

Over the last year, the number of participants who were employed in our study corresponded with the timing of the COVID-19 restrictions in BC (see Figure 3). Employment rates dropped within a month of the tightening of restrictions in April 2020 and increased with the easing of restrictions in May 2020.

Table 6 shows the number of missing responses from the baseline survey. Missing data was found in four of the outcome measures (GAD-7, PHQ-9, CPROM and MLT). There was no missing data in the SWLS. More data were missing from the demographic variables. Approximately 10% of data were missing from self-reported housing status and number of services used.

**Table 6:** Pattern of baseline missing data (*N* = 110).

**Variables**	***N***	**%**
Age	2	1.8
Ethnicity	10	9.1
Type of housing	11	10.0
Highest education	2	1.8
Primary diagnosis	9	8.1
Number of services used	12	10.9
Relapse of symptoms	9	8.1
Hospitalization	9	8.1
Talking to someone	9	8.1
Incomplete GAD7	2	1.8
GAD7 – Question 3	1	0.9
GAD7 – Question 8	4	3.6
PHQ – Question 9	1	0.9
Incomplete CPROM	2	1.8
MLT	1	0.9

## Discussion

The objectives of this study were to estimate the feasibility of the YESS program and the extent to which participation in the YESS program improved employment and mental health outcomes among youths with MHSU disorders. Our results showed that the YESS program was feasible, and ~33% of participants gained employment after the program and ~25% of the participants made improvements across all mental health outcomes, especially on outcomes measuring life satisfaction, and quality of life. However, due to the COVID-19 pandemic restrictions, only a small proportion of participants were employed after the 5-week program.

In general, on most outcome measures, more than 30% of participants made improvements with the biggest proportion reported on the SWLS and the MLT. The main outcome measure of the YESS program was obtaining employment, and only 33.3% of people secured employment at the end of the follow-up period. Due to the pandemic, certain industries (e.g., entertainment, animal care) were shut down and job opportunities were limited. Thus, unemployment rates were high and participants were competing with other more qualified candidates for the few available jobs, leading to decreased opportunities. In addition, some participants were hesitant to work during the pandemic. Most participants were youths with depression and anxiety, which were heightened during the pandemic. As such, many participants wanted to ease into employment and make sure they could handle the workload and being in a new environment, while others were happy to attend courses and training during this period.

The effectiveness of the YESS program could also be associated with the outcomes used in this study. Appropriateness of the outcome measures was assessed as part of implementation. Many of the outcome measures were moderately to highly correlated with each other. The strongest association was found between the GAD-7 & PHQ-9 (0.78) and the CPROM and MLT (0.76). Consistent with literature, strong associations between scores on the PHQ-9 and the GAD-7 were reported indicating a strong convergent validity between the two scales but this could also indicate that the measures may be measuring the same underlying construct (31, 32). While the constructs of anxiety and depression are conceptually distinct, it is difficult to differentiate between symptoms of depression and anxiety and these two conditions often co-occur in many mental health conditions (33). Thus, even though they are two separate scales, it is perhaps best to use both screening scales at the same time for a more accurate picture of the individual's psychological distress. However, both the PHQ-9 and the GAD-7 are screening tools and therefore, would be more useful as tools to screen participants at baseline and using the information to help the team better plan and recommend the appropriate treatment strategies to manage their depression and anxiety.

The high association between the CPROM and the MLT was also expected, as some items on the CPROM like general wellbeing are similar to the MLT. The CPROM measures the construct of recovery while the MLT measures quality of life and these two constructs are very closely associated in mental health research (34). Interestingly, there were no strong associations related to employment, indicating that gaining employment for people with mental health was not associated with a higher quality of life or recovery and may be largely due to external factors (11, 35).

At the end of the 5 and 16 weeks, about 20–30% of participants made improvement. Results showed improvements on the self-reported measures like a decrease in depressive and anxiety symptoms (36, 37). While this is still an encouraging result for a short intervention program, the majority of participants did not achieve the same level of clinical improvement. This could be due to a few reasons; the measures were not sensitive enough to detect change or the time between measurements may not have been long enough for change to occur. Both reasons are likely. Firstly, the YESS program consisted of workshops on career building skills and psychiatric services like peer support. Given the intervention provided in our study, it is unlikely that the intervention would impact quality of life or recovery directly after a short intervention, quality of life and recovery would seem more like a downstream effect of improvement of symptoms and gaining employment (38, 39). Therefore, it is more ideal to measure recovery and quality of life after participants have demonstrated better symptom management and/or a change in employment status. Lastly, there has been some evidence that both the PHQ-9 and the GAD-7 are not sensitive enough to detect change over time at an individual level (40, 41). As such, modified versions of the PHQ-9 and the GAD-7 have been suggested through modern measurement analysis (41, 42). To estimate the efficacy of the YESS program more accurately and measure individual change over time, measures with increased sensitivity should be considered. Another possible approach would be to use an individualized measurement approach to measure effectiveness of SEP where participants list and rate the top priority areas that they wish to achieve from participating in a SEP. In summary, a broader approach to the measurement of outcomes of the IPS programs should be used (43, 44).

Other aspects of feasibility were also examined. The study commenced in November 2019 and a total of 110 participants were recruited at the end of recruitment in December 2020. All eligible participants who were interested in the program were recruited. The participants in this study were from diverse backgrounds and more than 50% identified as women. Despite the COVID-19 pandemic, the demand in this program remained consistent throughout the study period. While recruitment was affected by the initial COVID-19 restrictions, the number of participants recruited steadily increased until December 2020, indicating that there is a demand for the program among youths in BC. The switch to online recruitment could also have helped maintain recruitment rates. Online recruitment appears to be an effective way to recruit people and reach out to people in need of services during the pandemic. However, all responses to our online advertisement had to be screened as not everyone met the eligibility criteria. This demand for our program could also be driven by the COVID-19 pandemic. Based on baseline results, our participants reported moderate depression and anxiety, and most were slightly dissatisfied with their lives. For people with existing mental health issues, the impact of this pandemic might be more acute and therefore extra efforts should be made to ensure that there is continued access to mental health and employment services (45).

The retention rate of the YESS program was ~75%, with an attrition rate of 25%, which is a better result than most youth mental health programs where the average drop-out rate of youth mental health programs ranged from 28% to 78% (46). The proportion of women who completed the program was higher, highlighting the possible gender difference in retention rates in mental health programs (47, 48). In addition, a higher proportion of non-completers of our program were in at-risk housing and people who accessed multiple services. This is consistent with current literature where youths who experienced difficulties in other aspects of life like homelessness were more likely to drop out of mental health programs (49). These results suggest that completion of SEP may be dependent on one's housing situation. For future expansion of the YESS program, additional support on securing income assistance and housing should be included or as a prerequisite (50). Employing a more person-centred approach, like having an individualized treatment plan, to address the various issues that might be influencing one's ability to gain employment may also help overcome these challenges (51).

In terms of practicality of the program, patterns of missing data were examined. Missing data was more prevalent with demographic variables than outcome measures. More than 10% of data was missing from demographic variables on housing situation and number of services used. There were also more missing data on questions related to diagnosis, symptoms, and hospitalization. This might be due to the sensitive nature of the questions. For people who are in at-risk or precarious housing situations, their situation might be constantly evolving, making it difficult to answer questions on their housing situation. Questions regarding diagnosis and symptoms might also be difficult to answer and many youths often suffer from more than one mental disorder at any one point in time (52). To increase practicality and integration of the program, answers to questions relating to health condition could be modified by using medical records or asking participants to list most bothersome symptoms. Listing most bothersome symptoms would also be more in line with an individualized measurement approach. An initial interview with the participant could also allow us to know more about the participant's living situation and health condition. Additionally, it is important to note that despite the COVID-19 restrictions on in-person meetings, we were also able to run the YESS program virtually after the COVID-19 restrictions were imposed. This further reflected the adaptability of the YESS program structure and format.

### Limitations

Some limitations existed in this study. As the YESS program was unique to one Canadian urban centre, transferability of the study findings is limited to this context. Additionally, due to the COVID-19 pandemic, recruitment took a longer than expected time. We also did not collect data on participants' diagnoses and medication, which would have given us a clearer picture of the description of participants and the relationship between these factors and employment. Qualitative data was also not collected in this study due to the uncertainty around the COVID-19 pandemic and emerging ethical protocols for how to conduct one on one interviews. Qualitative data from participants and members of the team would have helped to better understand experiences in the program, and feasibility outcomes.

## Conclusion

Overall, the results showed that the YESS program was feasible, even within the context of the global COVID-19 pandemic. However, employment, as for many youths across Canada, was difficult to achieve. Engagement in this program brought about unexpected benefits that go beyond employment such as improved mental health and recovery outcomes. An individualized approach to treatment and measurement is recommended for future implementation of the YESS program. Results of this study have affirmed that a supported employment program can be integrated and implemented on a larger scale within an integrated youth service network in BC.

## Data availability statement

The original contributions presented in the study are included in the article/supplementary material, further inquiries can be directed to the corresponding author/s.

## Ethics statement

The studies involving human participants were reviewed and approved by the University of British Columbia's Behavioral Research Ethics Board (H21-01510). Written informed consent for participation was not provided by the participants' legal guardians/next of kin because: Written informed consent is needed only for children aged 14 years and below in British Columbia, Canada. We did not have any participants who were minors and therefore written consent from legal guardians was not required. All participants were 17 years old and older and able to provide written consent independently.

## Author contributions

NO: conceptualization, data curation, formal analysis, methodology, project administration, investigation, and writing-original draft. KM and KG: writing-review and editing and visualization. DA: conceptualization, data curation, and writing-review and editing. SM: conceptualization, funding acquisition, and resources. SB: conceptualization, funding acquisition, methodology, validation, resources, supervision, project administration, and writing-review and editing. All authors contributed to the article and approved the submitted version.

## Funding

This study was funded by Canadian Institute Health Research Project (Grant Number: 156191).

## Conflict of interest

The authors declare that the research was conducted in the absence of any commercial or financial relationships that could be construed as a potential conflict of interest.

## Publisher's note

All claims expressed in this article are solely those of the authors and do not necessarily represent those of their affiliated organizations, or those of the publisher, the editors and the reviewers. Any product that may be evaluated in this article, or claim that may be made by its manufacturer, is not guaranteed or endorsed by the publisher.
